# Up-regulation of SERPINA3 correlates with high mortality of melanoma patients and increased migration and invasion of cancer cells

**DOI:** 10.18632/oncotarget.9409

**Published:** 2016-05-17

**Authors:** Jiaying Zhou, Yabin Cheng, Liren Tang, Magdalena Martinka, Sunil Kalia

**Affiliations:** ^1^ Faculty of Science, University of British Columbia, Vancouver, BC, Canada; ^2^ School of Pharmaceutical Sciences, Xiamen University, Xiamen, China; ^3^ Department of Dermatology and Skin Science, University of British Columbia, Vancouver, BC, Canada; ^4^ Welichem Biotech Inc, Burnaby, BC, Canada; ^5^ Department of Pathology and Laboratory Medicine, University of British Columbia, Vancouver, BC, Canada

**Keywords:** SERPINA3, melanoma, AACT (alpha-1 antichymotrypsin), tissue micro array, cell migration and invasion

## Abstract

Serpin Peptidase Inhibitor, clade A member 3 (SERPINA3) was found to be abnormally overexpressed in a subset of melanoma tissue biopsies. High SERPINA3 expression was also associated with poor patient survival. In this study, we set out to test SERPINA3 protein's prognostic potential with a larger-sized and independent patient cohort, and to explore SERPINA3's function in melanoma cells. Tissue microarray-based immunohistochemistry analysis showed a significant increase in SERPINA3 expression in invasive and metastatic melanomas compared to normal nevi and melanoma-in-situ (*P* < 0.001, Chi-square test). In melanoma patients, high SERPINA3 expression was strongly associated with worse overall and disease specific survival at 5 years. Multivariate Cox regression analysis showed that SERPINA3 expression is an independent prognostic factor to predict melanoma patient clinical outcome. When SERPINA3 expression was selectively silenced using small interfering RNA molecules (siRNA) in cultured melanoma cell lines, cell migration and matrix invasion was significantly decreased, but no change in cell proliferation was observed.

This study confirms the prognostic potential of SERPINA3 expression in human cutaneous melanoma and reveals the pro-migration and pro-invasion functions of this protein on melanoma cells.

## INTRODUCTION

Melanoma is an aggressive cancer that arises from the abnormal growth of pigment-producing skin cells. Although accounting for only 2% of all skin cancer cases in North America and demonstrating high recovery rates with early stage surgical excision, melanoma is the most aggressive skin cancer with high patient mortality (> 80%) after becoming metastatic [1, 2]. Recent breakthrough in metastatic melanoma treatment using the MAP Kinase inhibitors and the checkpoint antibodies has significantly changed the landscape of melanoma therapy; however, both regimens have their specific limitations. Currently, metastatic melanoma is still a fatal disease, with a median survival time of 6 to 10 months [3]. Therefore, identifying the mechanism that is responsible for melanoma progression and metastasis may provide insights into the design of effective and therapeutic strategies for melanoma.

High throughput gene expression profiling studies have uncovered multiple genes involved in melanoma progression and metastasis [4–9]. Some of the striking changes in metastatic melanoma were identified in genes encoding proteins involved in the regulating and remodeling of the extracellular matrix (ECM), such as osteopontin (OPN), collagen triple helix containing protein 1 (CTHRC1) [10, 11], and serpin peptidase inhibitor, clade A (alpha-1 antiproteinase, antitrypsin), member 3 (SERPINA3) [12, 13]. SERPINA3 (previously known as α1-antichymotrypsin) is a 68kDa secreted serine protease inhibitor normally produced by the liver that proteolytically inhibits the activity of several serine proteases including chymotrypsin and cathepsin G [14]. This acute phase reactant protein is involved in a wide range of physiological activities such as blood coagulation, complement activation, apoptosis, wound healing, and embryonic development [15–17]. Recently, up-regulated SERPINA3 expression has been reported in multiple cancer types, including cancers of the colon [18], breast [19–21], prostate [22–25], and stomach [26]. Specifically, SERPINA3 expression has been demonstrated to positively correlate with worsening prognosis in patients with breast, lung [27–29], and gastric cancers [30]. An early report revealed that the serum of SCID (Severe Combined Immunodeficient) mice carrying human metastatic melanoma cell lines had increased levels of SERPINA3; SERPINA3 also presented in 10 out of 17 melanoma biopsies within a large cancer pathology series [12].

Preliminary analysis on 216 patient tissue samples by our group showed that melanoma patients with high expression of SERPINA3 have shorter disease specific survival, suggesting that SERPINA3 expression could serve as a prognostic marker in melanoma [13]. To further investigate the role of SERPINA3 in melanoma pathogenesis and prognosis, we used tissue microarray (TMA) containing 411 melanocytic lesions (including 25 normal nevi, 20 melanoma in situ, 228 primary melanomas and 138 metastatic melanomas) and immunohistochemistry to evaluate the expression of SERPINA3 and its relation to clinicopathologic factors and patient survival. Our data indicated that up-regulation of SERPINA3 is significantly associated with melanoma progression and worse patient survival. In-vitro cell-based assays demonstrated enhanced cell migration and invasion by high SERPINA3 expression, suggesting that SERPINA3 may play an important role in melanoma progression.

## RESULTS

### Clinicopathologic features of TMAs

The TMAs used in this study consisted of a total 713 melanocytic lesions, all from patients of Caucasian descent. Because of biopsy cores loss and insufficient cells present in some samples, 366 melanomas (228 primary invasive melanomas and 138 metastatic melanomas), 20 melanomas in situ and 25 normal nevi were evaluated for SERPINA3 staining. The demographics and clinicopathologic characteristics of melanoma patients are shown in Table 1.

**Table 1:** Patient demographics

Variables	Total No.	SERPINA3		
		Low No. (%)	High No. (%)	*P*^†^
**All melanomas**	366			
Age (years)				
≤ 60	195	87 (45)	108 (55)	0.0636
> 60	171	60 (35)	111 (65)	
Sex				
Male	216	95(44)	121(56)	0.0738
Female	150	52(35)	98(65)	
AJCC stage				
I	109	60(55)	49(45)	0.0003*
II	119	45(38)	74(62)	
III	94	24(26)	70(74)	
IV	44	18(41)	26(59)	
**Primary melanomas**	228			
Age (years)				
≤ 60	114	59(52)	55(48)	0.0841
> 60	114	46(40)	68(60)	
Sex				
Male	120	65(54)	55(46)	0.0096*
Female	108	40(37)	68(63)	
Tumor thickness (mm)				
≤ 2.00	118	64(54)	53(46)	0.0085*
> 2.00	110	41(37)	69(63)	
Ulceration				
Absent	166	84(51)	82(49)	0.0378*
Present	60	21(35)	39(65)	
Site*				
Sun-protected	55	27(49)	28(51)	0.9025
Sun-exposed	162	78(48)	84(52)	
Unspecified	1			
Subtype				
Superficial spreading	90	48(53)	42(47)	0.0682
Lentigo maligna	42	12(29)	30(71)	
Nodular	30	15(50)	15(50)	
Others	25	9(36)	16(64)	
Unspecified	41	21(51)	20(49)	
**Metastatic melanomas**	138			
Age (years)				
≤ 60	81	28(35)	53(65)	0.2085
> 60	57	14(25)	43(75)	
Sex				
Male	96	30(31)	66(69)	0.7530
Female	42	12(29)	30(71)	

†: Chi-square test.

*: Sun-protested sites: trunk, arm, leg and feet; Sun-exposed sites: head and neck.

Patient Demographics with age and sex based on NN (Normal Nevi), MIS (Melanoma in Situ), PM (Primary Melanoma) and MM (Metastatic Melanoma) types of sample. AJCC Stage is a system devised by the American Joint Committee on Cancer to determine severity of tumors in patients. N/A denoted for data not available. Asterisks indicate significant p-values at α = 0.05.

For the total of 366 melanoma patients, there were 216 men and 150 women, with age ranging from 7 to 95 years (median age is 60 years). For the 228 primary melanomas, 118 tumors were ≤ 2.0 mm in thickness, and the other 110 were > 2.0 mm thick. Ulceration was observed in 60 cases. Fifty five primary melanomas were located in sun-exposed areas (head and neck), whereas 162 were located in sun-protected sites (other locations including trunk, arm, leg and feet). One hundred and thirty eight melanoma metastases were available for SERPINA3 staining evaluation, including 96 men and 42 women. According to the American Joint Committee on Cancer (AJCC) staging system, 109 and 119 cases were Stage I and II, respectively; 94 and 44 cases were Stage III and IV, respectively (Table 1).

### Increase of SERPINA3 expression correlates with melanoma progression

**Figure 1:**
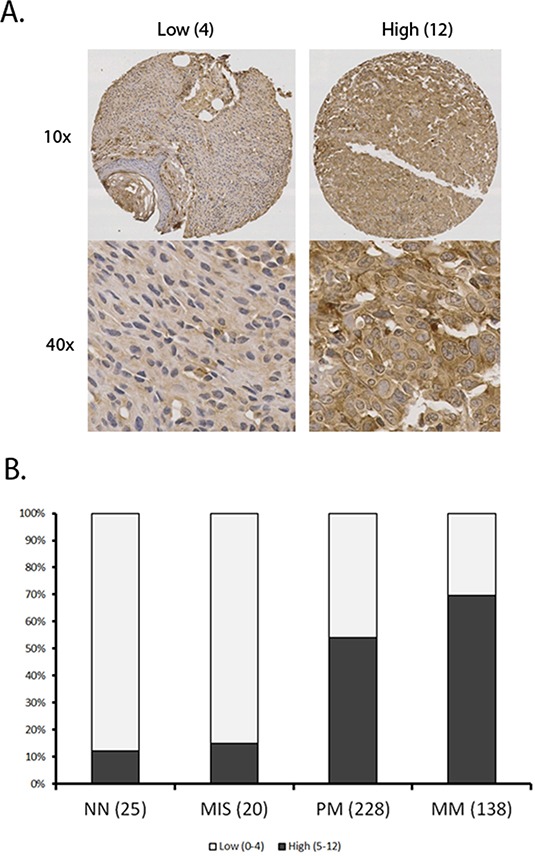
Summary of SERPINA3 staining data in various kinds of melanocytic tumor A tissue microarray was stained using immunohistochemistry and scanned. All samples were scored using a previously described 12 point scale involving intensity and percentage of melanoma cells showing staining for SERPINA3 (see materials and methods). Panel **A**. Representative images of tissues with high and low scores. Panel **B**. Increased SERPINA3 expression correlates with progression of melanoma. Sample sizes of each group are shown in bracket. A marked increase in percentage of samples with high SERPINA3 expression was shown between MIS and PM (*P* < 0.0001) and further between PM and MM (p<0.0001). NN, Normal nevi; MIS, Melanoma in situ; PM, Primary melanoma; MM, metastatic melanoma.

To investigate the expression level of SERPINA3 in the biopsies of pigmented lesions, we performed immunohistochemistry staining of normal nevi, melanoma in situ, primary melanoma and metastatic melanoma cores on TMA slides (Figure 1). The staining was predominantly in cytoplasm, and therefore only cytoplasmic staining was evaluated. Strikingly, we found marked increase of SERPINA3 expression in primary invasive melanomas (PM) compared with normal nevi (NN) and melanomas in situ (MIS) (*P* = 6.8E-5 and 8.4E-4, respectively, Chi-square test). A further increase was observed in metastatic melanoma (MM) compared with NN and MIS (*P* = 6E-8 and 2.4E-6, respectively, Chi-square test). Moreover, the staining of SERPINA3 was also increased in MM compared to PM (*P* = 3.1E-3, Chi-square test). This result indicates that SERPINA3 could play a critical role in melanoma initiation and progression process.

### SERPINA3 expression in melanoma and clinical parameters

**Figure 2:**
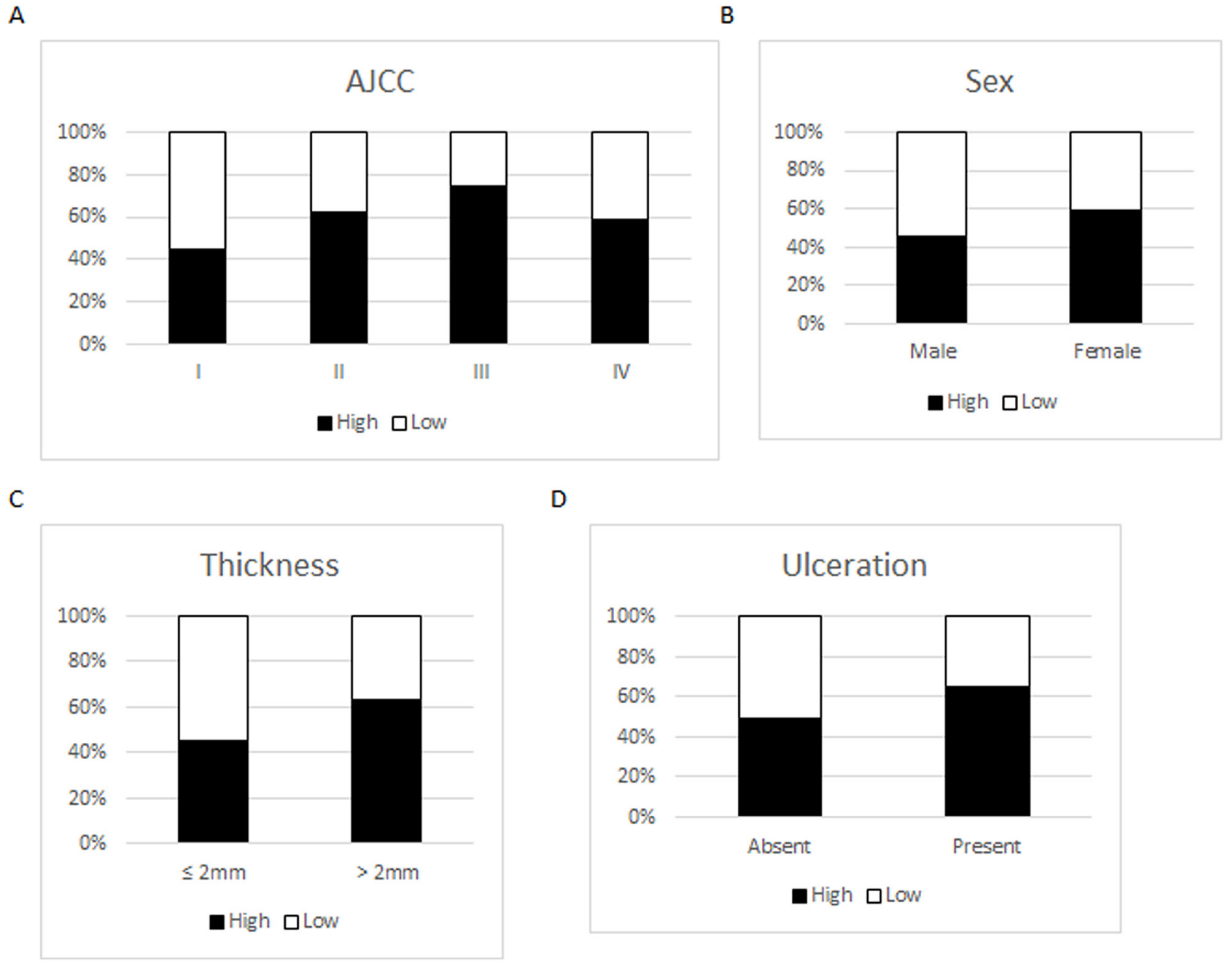
Expression of SERPINA3 in melanoma biopsies grouped according to various clinicopathologic paramaters Scoring scale: Low=0-4; High=5-12. Panel **A**. SERPINA3 expression grouped by AJCC stages. Panel **B**. SERPINA3 expression grouped by gender. Panel **C**. SERPINA3 expression grouped by melanoma thickness. Panel **D**. SERPINA3 expression grouped by ulceration status.

In the 366 melanoma cases, we found that high SERPINA3 expression ratio was significantly increased from 45% in AJCC Stage I to 62% in Stage II, and further increased to 74% in Stage III (*P* = 9.1E-3 and 2.0E-5, respectively, Chi-square test). The expression of SERPINA3 was slightly decreased in AJCC Stage IV compared to Stage III. However, this reduction is not statistically significant (*P* = 0.0673, Chi-square test) (Figure 2). Interestingly, we also observed stronger staining of SERPINA3 in female patients compared to male (*P*= 9.6E-3, Chi-square test). The reason for the different SERPINA3 expression levels between men and women is largely unknown and worth further investigation. A significant increase of SERPINA3 expression in melanomas thicker than 2 mm compared to that in thinner tumors was also detected (*P* = 8.5E-3, Chi-square test), which is further evidence that SERPINA3 could be important in melanoma progression. Not surprisingly, higher SERPINA3 expression (65%) was found in ulcerated melanomas compared to that in ulceration absent tumors (46%) (*P* = 0.0378, Chi-square test). We did not detect significant correlations between SERPINA3 expression and other clinicopathological variables, including age, location or subtypes (Figure 2).

### SERPINA3 expression in melanoma biopsies is correlated with poor patient survival over 5 years

**Figure 3:**
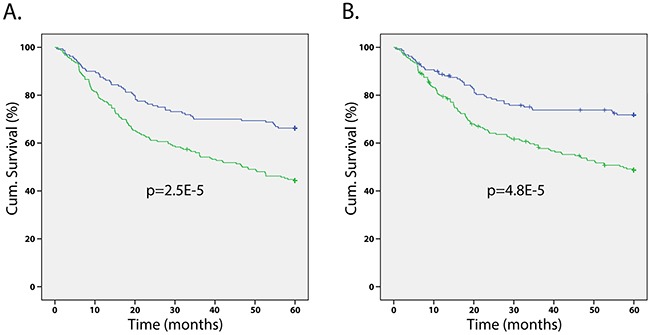
Correlation of SERPINA3 expression levels and patients’ survival Kaplan-Meier survival analyses on melanoma patients reveals that patients with high SERPINA3 (green line) expression have significantly worse **A**. overall and **B**. disease-specific 5-year survival than those with low SERPINA3 expression. (*P* < 0.0001 for both).

In order to determine the prognostic value of SERPINA3, Kaplan-Meier survival tests were run for the 5 year overall survival and disease-specific survival of melanoma patients. Kaplan-Meier survival tests determined that patients with high SERPINA3 expression had significantly lower 5 year overall and disease-specific survival (overall *P* < 0.0001, KM; disease specific *P* < 0.0001, KM) (Figure 3). Patients with higher SERPINA3 expression have a survival rate of approximately 40%, while patients with lower SERPINA3 expression have a survival rate of approximately 80%. A similar trend has been found in a previous study for 5 year survival of patients [13].

Analysis was also performed with patients separated into those with primary melanomas and those with metastatic melanomas (data not shown). While significant differences could still be seen, it was not significant at all stages, possibly due to relatively small case number for specific stages. Data is presented in this study using all melanoma cases; future studies will strive to clarify SERPINA3's correlation with survival at all stages.

Cox regressions were used to determine the independence of SERPINA3 as a prognostic marker from other factors in melanoma patient survival. For both overall and disease specific survival at the 5 year level, SERPINA3 was determined to be a significant factor of patient survival (overall *P* = 0.004, disease-specific *P* = 0.001), independent of AJCC stage, age, or sex of patients (Table 2).

**Table 2:** SERPINA3 as an independent prognostic marker for melanoma

	Overall Survival		Disease-specific Survival	
Parameters	HR	P	HR	P
AJCC Stage	2.590	0.000*	2.822	0.000*
Age	1.654	0.015*	1.239	0.332
Sex	1.077	0.731	1.018	0.940
SERPINA3	1.881	0.004*	2.280	0.001*

Cox regression of several clinical parameters in melanoma patients. AJCC (American Joint Committee on Cancer) staging is used to determine severity of tumors. HR, hazard ratio, indicates how much each factor increases mortality of patients. P indicates the p-value of each factor having an independent effect on mortality of patients. Asterisks indicate significant p-values at α = 0.05.

### SERPINA3 promotes melanoma invasion and migration in extracellular matrix without affecting cell proliferation

**Figure 4:**
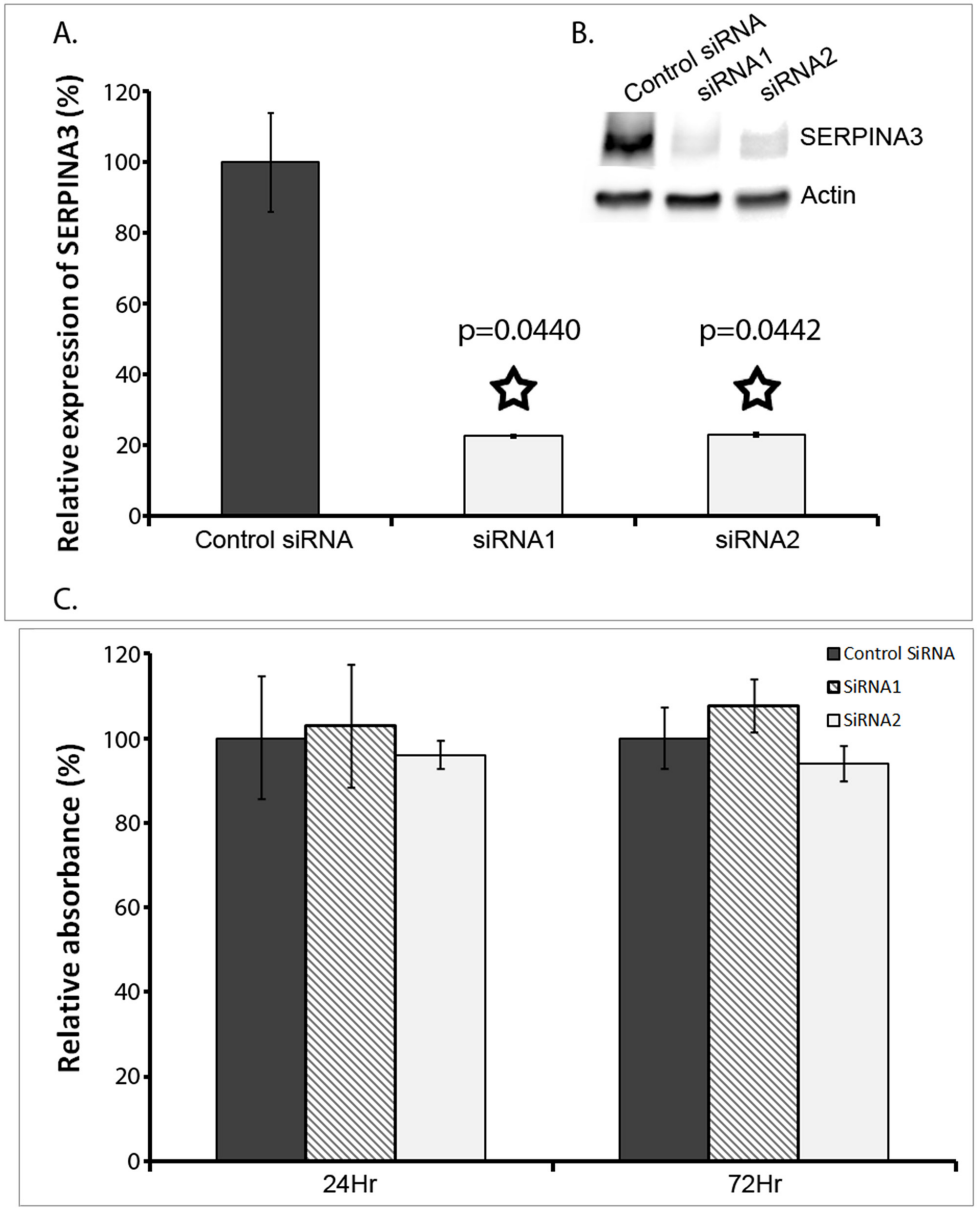
Effect of SERPINA3 Silencing on Melanoma Cell Proliferation Panel **A**. Knockdown of SERPINA3 expression in MMRU cells using synthetic small inhibitor RNA (SiRNA). Realtime PCR verified that SERPINA3 expression was significantly decreased in cultures cells transfected with two individually targeted SiRNA (SiRNA1 and SiRNA2) compared to random scrambled negative control SiRNA. Data represented as mean expression as compared to control +/− standard deviation. A Western blot is also shown from 72hrs after siRNA knockdown. Actin is used as the internal control, and SERPINA3 band seen at ~75kDa is shown. C: control SiRNA, 1: SiRNA1, 2: SiRNA2. Panel **B**. A Western blot is also shown from 72hrs after siRNA knockdown. Actin is used as the internal control, and SERPINA3 band seen at ~75kDa is shown. C: control SiRNA, 1: SiRNA1, 2: SiRNA2. Panel **C**. Effect of SERPINA3 silencing on melanoma cell proliferation, measured by MTS assay, represented as mean absorbance +/− standard deviation.

To examine the roles SERPINA3 overexpression plays in biological behaviors of melanoma cells, the cultured cell line MMRU was used as an *in vitro* cell model. This cell line was derived from a metastatic melanoma and had high intrinsic levels of SERPINA3 expression. By gene-specific siRNA mediated knock down, the expression of SERPINA3 was efficiently blocked at both mRNA and protein levels. As shown in Figure 4A, when compared to the control siRNA, both anti-SERPINA3 siRNA (siRNA1 and siRNA2) efficiently decreased the expression of SERPINA3 in MMRU cells. The knockdown efficiency measured by mRNA expression is 84% by siRNA1 and 88% by siRNA2. Similar knockdown efficiency is observed by Western blot protein analysis.

SERPINA3 was reported to play a role on migration of endometrial cancer cells [31]. The dramatic increase of SERPINA3 expression during the transition from benign or non-invasive lesions to invasive and metastatic tumors suggests that SERPINA3 is potentially involved in the development of invasiveness in advanced stages of melanoma. Therefore, the invasive and migration behaviors of SERPINA3-suppressed cells and cells transfected with control siRNA were studied through three different tests, explained below.

Cell proliferation was measured using MTS assay by comparing the effect of SERPINA3 siRNA knockdown in MMRU melanoma cells. As shown in Figure 4B, there was no significant difference in cell proliferation with siRNA knockdown, at both the 24hr mark and 72hr mark.

**Figure 5:**
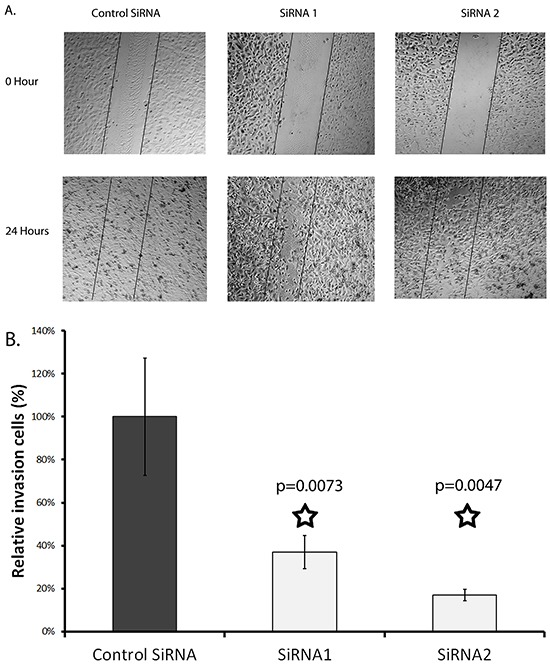
Effect of SERPINA3 Silencing on melanoma cell migration Panel **A**. Photomicrographs of representative scratch assay results. MMRU melanoma cells were transfected with SiRNA for 24 hours then re-seeded in 24 wells for scratch assay. Panel **B**. Migrating cells were counted in representative areas of each scratch 24 hours after scratching, represented as mean cell count +/− standard deviation.

A wound-healing assay was run to determine the effect of SERPINA3 knockdown on cell migration. It was found that siRNA knockdown significantly decreased MMRU cell migration, with less cells migrating into the gap formed in a scratch assay (Figure 5). Both siRNA knockdowns had a significant effect on cell migration, inhibiting cell migration by 67% and 73%, respectively.

**Figure 6:**
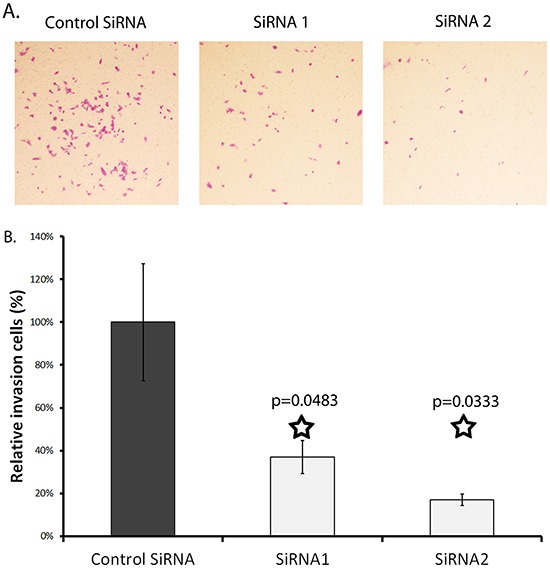
Effect of SERPINA3 knockdown on matrix invasion by melanoma cells Panel **A**. Photomicrographs of representative invasion cells. MMRU melanoma cells were transfected with SiRNA for 24 hours then seeded in metrigel coated Boyden chamber (8 μm pore size) in 0.1%FBS media; lower chambers contained 20%FBS media. Membranes were harvested at 24 and 48 hours by swabbing the gel off with cotton swabs and fixing cells that had invaded to the bottom of the membrane using formaldehyde and staining with Toluylene red. Panel **B**. Effect of SERPINA3 knockdown on cell invasion. Cells were counted after fixing and staining. Represented as mean cell count +/− standard deviation.

An in vitro Matrigel invasion assay was run to test the effect of SERPINA3 knockdown on cell invasion. Matrigel is a semi-solid protein mixture closely mimicking the extracellular matrix. The number of cells that invaded through the Matrigel was dramatically decreased when SERPINA3 protein expression was suppressed in MMRU cells through either siRNA1 (by 20%) or siRNA2 (by 50%) (siRNA1 *P* = 0.048, siRNA2 *P* = 0.033) (Figure 6).

## DISCUSSION

Proteolytic degradation of the ECM is considered an essential step in the invasion and metastasis of malignant cell to distant tissues [32, 33], and proteases expressed by neoplastic and/or stromal cells are therefore considered as key players in this process, such as matrix metalloproteinases (MMPs). As a consequence, protease inhibitors are intuitively expected to have an anti-malignant role [34, 35]. Paradoxically, some serine protease inhibitors (serpins) have been reported to be overexpressed in many tumor types [36], indicating a potential role in tumor progression.

As one of the most abundant serpins in human plasma, SERPINA3 has shown overexpression in many types of tumor [18–22, 26–28, 30]. In this study, we examined the expression pattern of SERPINA3 in a wide spectrum of melanocyte-derived tumor representing the various stages of melanoma progression, including benign nevi, melanoma *in situ*, invasive primary melanoma, and metastatic melanoma. SERPINA3 expression levels appear to strongly correlate with melanoma invasion and metastasis. Indeed, little SERPINA3 expression was detected prior to the development of invasive behavior. Melanoma *in situ* represents the radical growth phase (RGP) of melanoma in which malignant melanocytes are still confined within the epidermis and do not extend beyond the basement membrane; while invasive melanoma represents the vertical growth phase (VGP) in which melanoma cells invade through the basement membrane to reach the dermis or further invade into the subcutis. The transition from RGP to VGP is a biologically critical step in melanoma. Our findings suggest that SERPINA3 is involved in or related to the development of invasive behavior in melanoma cells, and thus associated with the progression of melanoma from RGP to VGP.

Further testing using Kaplan-Meier confirmed that patients with high SERPINA3 expression have lower survival rates, indicating possible clinical prognostic value of SERPINA3 in melanoma patients. Cox regression confirmed that SERPINA3 is an independent prognostic marker of patient survival, allowing for more specific prognosis of melanoma patients past the current AJCC system. Using SERPINA3 as a marker in addition to AJCC may allow for more accurate clinical prognosis, as well as a way to tailor treatment for specific patients.

Serpins have multiple complex roles in tumor biology. Although SERPINA3 has been shown to present in multiple tumors, its functional impact on tumor progression remains largely unknown. To further address this question, we performed *in vitro* analysis on cultured melanoma cell lines with siRNA-mediated down-regulation of SERPINA3 expression. Not surprisingly, down-regulation of SERPINA3 expression did not demonstrate an effect on the proliferation, survival and attachment of the melanoma cells (data not shown). This is consistent with the findings that SERPINA3 expression is largely absent in tissues of melanoma *in situ*, in which the melanocytes have already acquired their malignant proliferating properties. The interesting finding is that the ability of melanoma cells to invade through Matrigel was severely impaired with down regulation of SERPINA3 expression. Matrigel resembles the complex extracellular environment found in many tissues and is a validated *ex vivo* model of tissue matrix. It is suggested that SERPINA3 might promote melanoma invasion through remodeling the extracellular tissue matrix. While SERPINA3 may not be acting directly to induce this effect, knockdown of SERPINA3 is sufficient to decrease melanoma migration and invasion abilities. Therefore, molecules targeting SERPINA3 or maybe other serpins may have therapeutic implications for melanoma in the near future, although the precise mechanism of how SERPINA3, the serine protease inhibitor, affects the structure of ECM remains to be elucidated.

This study demonstrated that the up-regulation of SERPINA3 in advanced melanoma may contribute to the invasive behavior of melanoma cells by remodeling the extracellular tissue matrix. This finding may be useful in future prognosis of melanoma patients as well as a possible therapeutic target, and may lead to further understanding of melanoma and cancer progression as a whole.

## MATERIALS AND METHODS

### Ethics statement

Our study on archival melanoma biopsies was approved by the Clinical Research Ethics Board of the University of British Columbia. The experiments were performed in accordance with the Declaration of Helsinki guidelines.

### Tissue microarrays

The selection of melanoma tissue blocks and construction of tumor tissue microarrays have been described previously [10]. The formalin-fixed, paraffin embedded archival biopsies of benign melanocytic nevi, melanoma *in situ*, invasive primary melanoma and metastatic melanoma were obtained from the archival collection of Vancouver Coastal Health Authority's Pathology Department under the approbation of the University of British Columbia's Clinical Ethics Board (Vancouver, Canada).

Tissues with lost cores or insufficient cells were excluded from the study, leaving 411 tissues available for evaluation. The tissue microarray consisted of 25 normal nevi, 20 melanoma in situ, 228 primary melanoma, and 138 metastatic melanoma samples. All clinicopathological data was available for all melanoma cases. The biopsies were obtained from the 1990 to 2009 archives from the Department of Pathology at Vancouver General Hospital, Vancouver, Canada.

### Immunohistochemistry (IHC) staining of TMA

SERPINA3 protein expression was analyzed by immunohistochemistry on paraffin-embeded skin biopsies as described in previously published papers [37]. Briefly, deparaffinised 4μm tissue sections were treated 30 minutes in 0.1M sodium citrate (PH6.0) at 95 degree for antigen retrieval. Slides were then treated with 3% hydrogen peroxide for 30 minutes to block endogenous peroxidase activities. After blocking with protein block serum free solution (product # X0909, DAKO Corporation, Carpinteria, CA) Anti-SERPINA3 polyclonal antibody (product #A0022, Dako Corporation, Carpinteria, CA) was diluted in antibody diluent (product # S0809, DAKO Corporation, Carpinteria, CA) and applied to sections by incubation overnight in 4° Celsius. Normal rabbit serum with same concentration was used as a negative control. 10 μg/ml anti-S100 polyclonal antibody (product #Z 0311, Dako Corporation, Carpinteria, CA) was used on adjacent sections to locate melanocyte-derived cells. To visualize positive staining, we used EnVision + Dual Link System –HRP (product # K4063, Dako Corporation, Carpinteria, CA) followed by DAB Substrate-Chromogen System (product # K3468, Dako Corporation, Carpinteria, CA).

### Quantification of SERPINA3 staining intensity and statistic analysis

A previously described [10, 37] 4-point scoring system was used to determine intensity of SERPINA3 staining. Scoring was performed by three independent scorers, including a dermatopathologist, without access to clinico-pathological information of the sections. Discrepancies among the scorers were resolved by obtaining a consensus score, whereby the group evaluated the sections simultaneously using scanned microscope images. Section staining was evaluated using the 12-point Remmele scale [38]. Briefly, staining intensity was scored as 0 (negative), 1 (weak), 2 (moderate) and 3 (strong). The percentage of SERPINA3 expressing cells was scored into four categories: 1 (0–25%), 2 (26–50%), 3 (51–75%) and 4 (76–100%). In the cases with a discrepancy between duplicated cores, the higher score from the two tissue cores was taken as the final score. The level of staining was evaluated by immunoreactive score (IRS), which is calculated by multiplying the scores of staining intensity and the score of the percentage of positive cells. Based on the IRS, SERPINA3 staining pattern was defined as weak (IRS: 0–4) and strong (IRS: 6–12).

Statistical analysis was performed with GraphPad Prism 5 software (GraphPad Software, San Diego, CA). Differences in SERPINA3 staining in the various stages of melanoma were evaluated using chi-squared (χ^2^) analysis. The effects on cell apoptosis, proliferation, migration, and matrix invasion of cultured melanoma cells were evaluated using Student's t test. The statistical significance level was set at p<0.05.

Survival analysis was performed using SPSS software. Cytoplasmic staining was split arbitrarily. Kaplan-Meier tests were used to determine significance of cytoplasm SERPINA3 intensity on survival of patients. Cox regression was used to determine independence of SERPINA3 as a risk factor for melanoma patients.

### SERPINA3 RNA interference and quantification of expression levels

Melanoma cell line MMRU was a kind gift from Dr. G. Li of the University of British Columbia (Vancouver, Canada). Cells were cultured in Dulbecco's modified Eagle's medium (DMEM) (Hyclone, Logan, UT) supplemented with 10% fetal bovine serum (Hyclone, Logan, UT) at 37°C in 5% CO_2_ humidified atmosphere. The target short interfering RNA (siRNA) sequences of *SERPINA3* are:

5′- AAGGACCATTGTGCGTTTCAA-3′ (siRNA 1) and
5′-AAGGCTGTGCTTGATGTATTT-3′ (siRNA2) synthesized by Qiagen (Mississauga, ON, Canada). The control siRNA was purchased from Qiagen (AllStars Negative Control siRNA). For siRNA transfection, melanoma cells (MMRU) were seeded overnight prior to transfection in antibiotic-free DMEM medium containing 10% FBS in a 6 well plate with 50% confluence prior to transfection. The cells were transfected with 100 pmole of siRNA using Lipofectamine 2000 (Life Technology, Burlington, ON) following the manufactural protocol.

RNA was extracted from siRNA treated cells 24 hours after transfection using RNAeasy kit (Qiagen, Mississauga, ON, Canada). cDNA was synthesized using Superscript Vilo kit (Life Technology, Burlington, ON). Real time PCR was performed using SYBR Select Master Mix (Life Technology, Burlington, ON) on a StepOne Plus real-time PCR machine (Life Technology, Burlington, ON). SERPINA3 expression was measured using house-keeping gene *GAPDH* as a standard.

Primer sequences:

*SERPINA3* forward: CTTCACCAGCAAGGCTGACC
*SERPINA3* reverse: GCACAGCCTTATGGACCACC
*GAPDH* forward: AAGATCATCAGCAATGCCTCC
*GAPDH* reverse: TGGACTGTGGTCATGAGTCCTT
*SERPINA3* was measured as copies per 1000 copies of *GAPDH*. *SERPINA3* inhibition was expressed as a percentage of expression compared to the levels expressed in cells treated with control siRNA.

Protein was extracted from cells 72 hours after initial of siRNA treatment by lysing cells in RIPA protein lysis buffer containing protease inhibition cocktail (Roche, Indianapolis, IN) with 3 pulse of 5 seconds sonication. Protein concentration was measured using BCA protein assay kit (ThermoFisher Scietific, Rockford, IL). 30ug of protein per sample was loaded onto polyacrylamide gel and transfer to PVDF membrane after electrophoresis separation. The membrane was blocked with 5% bovine serum albumin in TBST buffer followed by overnight incubation in 4 degree with 2.5ug/ml of anti-SERPINA3 antibodies from rabbit (A0022, DAKO, Carpinteria, CA). Membrane was washed and treated with IRDye conjugated goat anti-rabbit IgG secondary antibody (Li-cor, Lincoln, NE). Same blot was then incubated with mouse monoclonal anti-β-Actin antibody from mouse (Sigma, Oakville, Ontario, Canada) followed by IRDye conjugated goat anti-mouse IgG secondary antibody. The membrane was then scanned using Li-Cor Odyssey® CLx imaging system.

### Cell proliferation assay

Proliferation of MMRU cells under siRNA knockdown of *SERPINA3* was performed by using an MTS assay. Cells were treated with control siRNA, siRNA1 or siRNA2. After treatment, cells were trypsinized and re-seeded in a 96-well plate with triplicates. Cells were then cultured for 24 hours and 72 hours in 37°C, 5% CO_2_ incubator before measuring viability bystaining with CellTiter 96 Aqueous One Solution Cell Proliferation Assay (Promega, Madison, WI) for 2-4 hours, then analyzed at 490nm using Epoch Microplate Spectrophotometer (Biotek, USA). A standard curve was made using a log of 2 serial titrating cells seeded at the same time with siRNA treated cells.

### Migration assay

To examine the migration of melanoma cells, a wound healing assay was performed. Cells were transfected with siRNA at 100 pmole in 6 wells for 24 hours then trypsinized and seeded in 24 wells with confluence density. After 4 hours of settlement, a sterile 200 μl pipette tip closed with flame was used to scratch the layer of cells. The detached cells were removed by gentle washing of PBS twice. Medium containing 1% FBS was replaced. The gaps were monitored using microscope and pictures were taken at same spot on time 0 and 24 hours after scratching to visualize cell migration activity. Each experimental group were repeated three times. Migrating cells were calculated after photographed.

### Matrigel invasion assay

The invasive behaviors of transfected cells were assessed by an in vitro Matrigel invasion assay [39] [40]. Briefly, cells were trypsinized 24 hours post siRNA transfection and seeded on top of a 8 μm pore size Boyden chamber insert membrane coated with Matrigel (BD Biocoat Metrigel invasion chamber, #354480, BD Biosciences, Palo Alto, CA) using a 24-well tissue culture plate. 5×10^4^ cells per well were seeded in 250 ul of DMEM medium supplemented with 0.1 % FBS. Chamber bottoms filled with 750 ul of 20% FBS-DMEM medium were used as a chemo-attractant. Cells were harvested at 24 and 48 hours. Cells on membrane surface were removed by swabbing three times with a cotton swab. Cells that had invaded to the other side of the membrane were washed twice with PBS, fixed with 4% formaldehyde in PBS for 30 minutes, and then stained with Neutral/Toluylene red or 0.1 % toluidine blue in PBS. After washing three times with water, the membranes were air-dried and mounted on glass slides. Slides were photographed under an inverted microscope. The assay was conducted twice.
